# Supplementary figures and images for: A novel imaging method (FIM-ID) reveals that myofibrillogenesis plays a major role in the mechanically induced growth of skeletal muscle
Source: eLife. 2024 Mar 11;12:RP92674. doi: 10.7554/eLife.92674 (PMC10928493; doi:10.7554/eLife.92674)

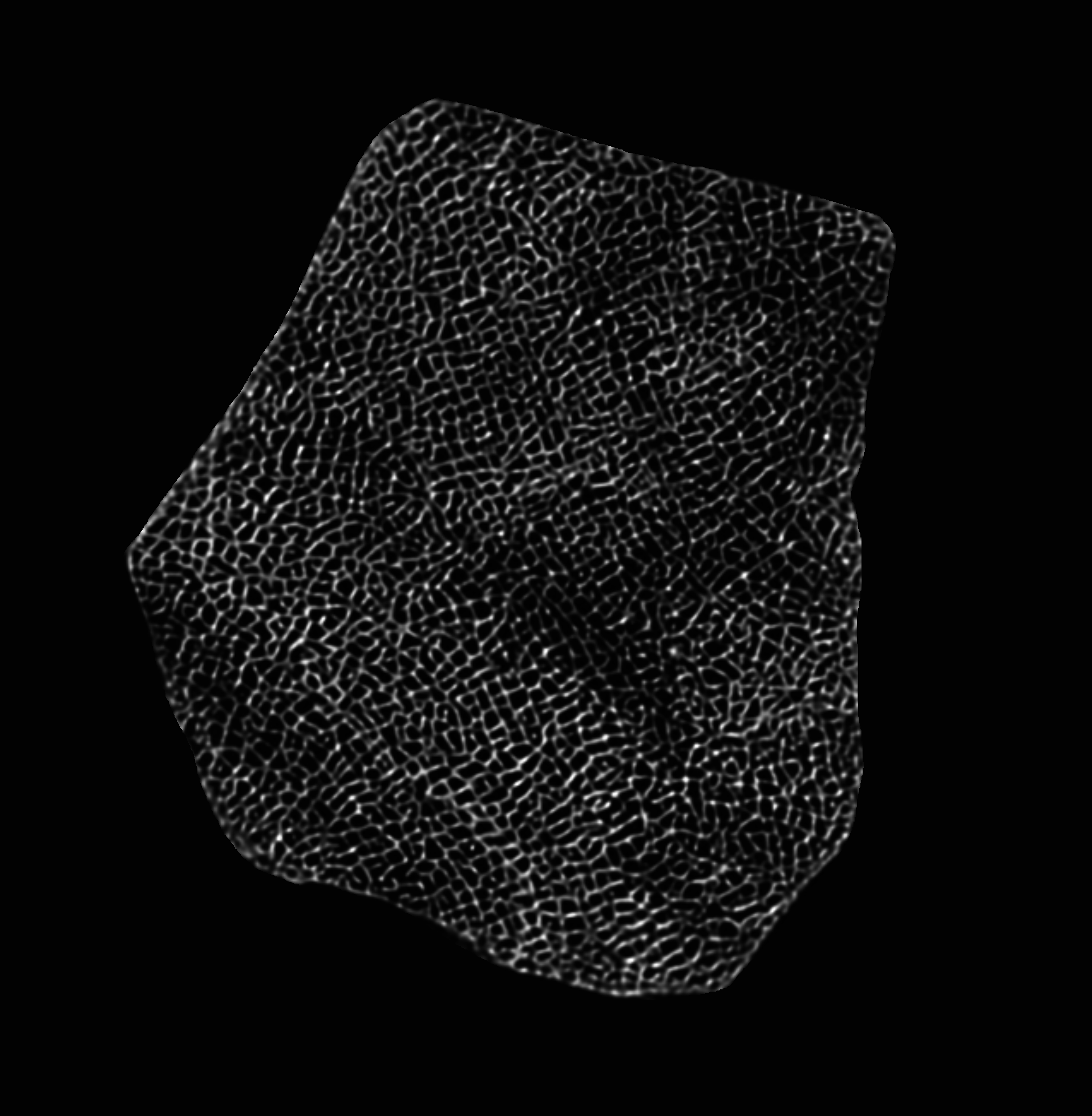

Supplement: Source code 1. [file elife-92674-code1.zip › CellProfiler Supplemental Files/Example Images/Ch0_Isolated_Fiber.tif]

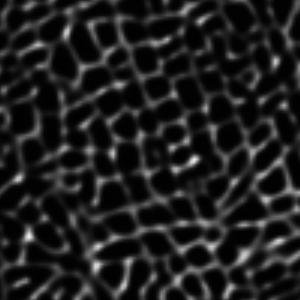

Supplement: Source code 1. [file elife-92674-code1.zip › CellProfiler Supplemental Files/Example Images/Ch0_ROI_of_Isolated_Fiber.tif]
